# Quantitative Determination of the Cytotoxic Compounds in Different Organs of *Arctium minus* (Hill) Bernh. by LC-HRESIMS Using Respond Survey Methodology

**DOI:** 10.1021/acsomega.4c06644

**Published:** 2024-09-25

**Authors:** Ebru Erol, Kubra Feyza Erol, Rabia Sare Yanikoglu, Cem Taskin, Cagla Kizilarslan Hancer, Gulacti Topcu

**Affiliations:** † Department of Analytical Chemistry, Faculty of Pharmacy, Bezmialem Vakif University, 34093 Istanbul, Türkiye; ‡ Department of Nutrition and Dietetics, Hamidiye Faculty of Health Sciences, University of Health Sciences, 34093 Istanbul, Türkiye; § Department of Biochemistry, Faculty of Pharmacy, Bezmialem Vakif University, 34093 Istanbul, Türkiye; ∥ Department of Pharmaceutical Botany, Faculty of Pharmacy, Bezmialem Vakif University, 34093 Istanbul, Türkiye; ⊥ Department of Pharmacognosy, Faculty of Pharmacy, Bezmialem Vakif University, 34093 Istanbul, Türkiye; # Drug Application and Research Center, Bezmialem Vakif University, 34093 Istanbul, Türkiye

## Abstract

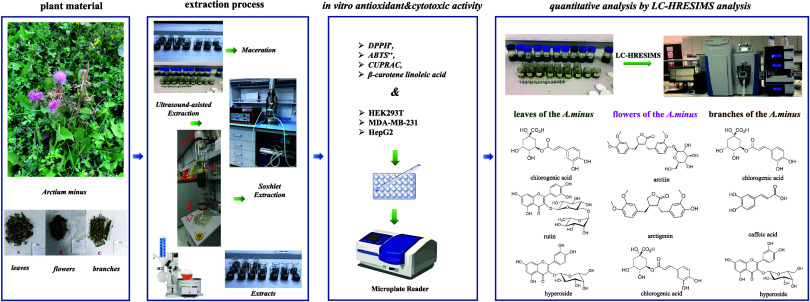

*Arctium minus* (Hill) Bernh., commonly known as “Burdock”, is a species within the *Arctium* genus of the Asteraceae family. Determining the optimum extraction conditions to obtain a concentrated extract with targeted active ingredients guides the most efficient use of natural products. Herein, ultrasound-assisted extraction (UAE) was optimized by using response surface methodology (RSM) to extract bioactive compounds from different organs of *A. minus*. Furthermore, phytochemical composition of extracts of *the A. minus* was investigated by using liquid chromatography-high resolution electrospray ionization mass spectrometry (LC-HRESIMS), antioxidant potential by using 2,2-diphenyl-1-picrylhydrazyl (DPPH), diazanium;3-ethyl-2-[(3-ethyl-6-sulfonato-1,3-benzothiazol-2-ylidene)hydrazinylidene]-1,3-benzothiazole-6-sulfonate (ABTS), CUPRAC, and metal chelation assays, and cytotoxic activities by using human breast cancer cell line (MDA-MB-231) and hepatocellular carcinoma cancer cell line (HepG2), and compared against conventional methods; Soxhlet and maceration. The RSM was employed to investigate the influence of ultrasound power, extraction time, and extraction temperature on the antioxidant potential assessed by the DPPH free radical scavenging assay. In UAE of *A. minus* leaves, flowers, and branches, the conditions resulting in the minimum IC_50_ values: 20 °C for 6 min at 50 W for leaves, 20 °C for 3 min at 100 W for flowers, and 20 °C for 3 min at 100 W for branches. Chlorogenic acid was identified as the major phenolic compound in the extracts obtained by UAE, with concentrations of 24,666.96 μg/g in leaves, 1054.92 μg/g in flowers, and 3,501.24 μg/g in branches. Flowers of *A. minus* had significantly higher levels of arctiin and arctigenin than those of leaves and branches. Extracts from leaves and flowers were more effective against MDA-MB-231 and HepG2 cancer cell lines than arctiin and arctigenin.

## 1 Introduction

World Health Organization reports that over 60% of developing countries use natural products to treat diseases such as cancer, diabetes, cardiovascular, and neurodegenerative diseases, indicating their widespread use. To provide new perspectives and solutions for treating these diseases, the search has begun for plant-derived, new, safe antioxidants, and anticancer molecules. The selection of the most accurate and reliable method for the qualitative and quantitative determination of these active molecules of the plant is an effective guide in using these active molecules in preclinical research and in identifying the chemical content of natural products. Response surface methodology (RSM) combines statistical and mathematical approaches to fit data to a polynomial model, allowing the behavior of all data to be analyzed and used to create a mathematical model for predictions.^1,2^

Conventional techniques, such as maceration, distillation, and Soxhlet extraction (SE), are commonly used to extract bioactive compounds from plant materials. However, these methods have significant drawbacks, including low extraction efficiency, excessive solvent usage, high energy consumption, and long processing times.^3^ Due to the limitations of traditional extraction methods, environmentally friendly techniques combined with modern technologies are gaining attention and are widely used by researchers. Green extraction methods aim to reduce solvent and energy use, increase extraction efficiency, and shorten processing times. One such method is ultrasound-assisted extraction (UAE), which meets all of these criteria. Ultrasound or ultrasonic wave is generally defined as frequency higher than 20 kHz, which is the human auditory perception threshold between 20 and 10.000 kHz.^4,5^ Nowadays, ultrasound-assisted extraction is used for the extraction of valuable bioactive compounds from various plant materials.

The species of the genus *Arctium*, also known as ’Burdock’, belongs to the Asteraceae family. They are biennial herbs found in wasteland, streams, and roadsides, less commonly in woods and forests, temperate regions of Europe and Asia, and sporadically in subtropical and tropical areas.^6^ There are three species in Turkey, including *Arctium minus* (Hill) Bernh., which is our study plant (Ekim, 2012). *A. minus* is known in English as “Common or Lesser burdock” and in Turkish as “Löşlek”.^7^

Considering the studies conducted on *A. minus*, here are a few examples of its traditional uses around the world. Karadeniz et al. investigated plants traditionally used by native Americans and identified *A. minus* as one of them. Additionally, it was noted that the roots are used as an antirheumatic, antipyretic, tonic, blood purifier, and to treat cough, abscesses, and abdominal pain.^8^ In Iran, the leaves and roots of *A. minus* have been traditionally used for blood purification, rheumatism, kidney ailments, skin diseases, and snake and scorpion stings.^9^ On the Iberian Peninsula, *A. minus* has been used against herpes, sores, scabies, and parasites.^10^ In Anglo-Saxon medicine, traditional preparations containing *A. minus* have been noted.^11^ In Portugal, *A. minus* is used for baldness and skin inflammation.^12^ Different parts of the plant are prepared and applied in various ways for different ailments. The aerial parts are used as tea by decoction or infusion for eye diseases. Additionally, the flowers are used by pounding or squeezing the juice for the same purpose. Its leaves are boiled with milk and used to treat swollen stomachs in children. The roots are pounded with milk or flour and applied to abscesses.^13^ Therefore, it is very important to determine the secondary metabolites contained in different organs of the plant. Notably, the biological activities and chemical composition of the various organs of *A. minus* have not been sufficiently studied, with only a few exceptions. In the study focusing on extracts from *A. minus* fruits, the chemical content was compared with that of other species, *Arctium lappa* and *Arctium tomentosum*, since the fruits of *A. minus* had not been studied before. Given that the composition of natural products can vary across different seasons and locations, the comparison of different species highlights the need for further research on the chemical content of *A. minus*.^14^ The total phenolic content of the extracts obtained from another subspecies of the *A. minus*, *A. minus* ssp. *minus* leaves, was examined as well as the anti-inflammatory and antinociceptive effects. Total phenolic results are 58.93 ± 2.72 mg/g in aqueous extract and 48.29 ± 0.21 mg/g in ethanol extract.^15^ The aerial parts of *A. minus* collected from Ireland were analyzed using UPLC-MS/MS, and the test results revealed a high amount of chlorogenic acid in the ethanol extract.^16^ The antioxidant, α-amylase, α-glucosidase, lipoxygenase, and tyrosinase enzyme inhibitions of different parts of *A. minus* roots were investigated. Also, the cytotoxic effects of extracts were investigated on MCF-7 and MDA-MB-231 breast cancer cell lines, in only one study.^17^ Herein, the aim of the present study was to assess the chemical composition and characteristics of polyphenolic extracts from different organs of *A. minus* using ultrasound-assisted extraction as an alternative technique in comparison to conventional methods such as maceration and Soxhlet extraction. First, all extracts were subjected to four different antioxidant tests: 2,2-diphenyl-1-picrylhydrazyl (DPPH), diazanium;3-ethyl-2-[(3-ethyl-6-sulfonato-1,3-benzothiazol-2-ylidene)hydrazinylidene]-1,3-benzothiazole-6-sulfonate (ABTS), CUPRAC, and metal chelation assays. Also, the cytotoxic potential of obtained extracts using the 3-(4,5-dimethylthiazol-2-yl)-2,5-diphenyltetrazolium bromide (MTT) method on the human healthy kidney cell line (HEK293T9), human breast cancer cell line (MDA-MB-231), and human hepatocellular carcinoma cell line (HepG2). In addition, the phenolic profiles of the extracts obtained by different extraction methods were analyzed using liquid chromatography-high resolution mass spectrometry (LC-HRMS).

## 2 Results and Discussion

### 2.1 Ultrasound-Assisted Extraction and Response Surface Methodology (RSM)

Experimental design RSM in ultrasound-assisted extraction (UAE) and mean values for the DPPH free radical scavenging assay of *A. minus* leaf are given in Table S5 (Supporting Information). Antioxidant activity results of the leaves extracts were given IC_50_ μg/mL value that found between 55.28 ± 2.21 and 161.13 ± 0.68 under different extraction conditions. While the maximum IC_50_ value was obtained at 20 °C, 6 min, and 50 W (R10), the minimum IC_50_ value was obtained at 40 °C, 6 min, and 150 W (R16). The regression correlation efficiency for the estimated model equation was determined as *R*^2^ = 0.9871 (Table S2). Moreover, since the *p*-value was less than 0.05 and the lack of fit value was greater than 0.05 (not significant), the quadratic model fit this study.

The predicted y response for the IC_50_ value of the extracts of *A. minus* leaves is expressed in terms of coded values by the following quadratic polynomial equation and eq 1 below:
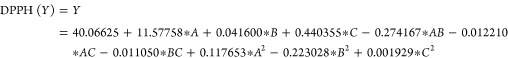
1

According to the analysis variance result, extraction temperature (*A*) (*p* ≤ 0.05), extraction time (*B*) (*p* ≤ 0.05), extraction power (*C*) (*p* ≤ 0.05), quadratic effect of extraction temperature (*A*^2^) (*p* ≤ 0.05), correlation effect of extraction temperature and time (*AB*) (*p* ≤ 0.05), and correlation effect of extraction temperature and power (*AC*) (*p* ≤ 0.05) were found to be significant.

The regression coefficient was calculated as *R*^2^ = 0.9871, and the corrected coefficient of determination was calculated as *R*_adj_^2^ = 0.9705. A model’s *R*^2^ value must be close to 1.00, the difference between the model’s *R*^2^ and corrected *R*^2^ values must be less than 0.2, and the coefficient of variation (CV) with its very low value of 5.56 indicates that the model is in acceptable fit for experimental data and is reliable.^18^

As seen in the equation regarding the DPPH value of *A. minus* leaves samples, the linear term (*A*) of the extraction temperature has a positive coefficient, which shows that the DPPH value increases with the increase in the extraction temperature. In addition, the fact that this factor has the highest coefficient value shows that it has the most significant effect on the DPPH value. Studies indicate that prolonging the extraction time may increase oxidation in the extracts, which may cause too much polyphenol loss. It has also been reported that there may be a decrease in their ability to capture free radicals resulting from the breakdown of phenolic compounds.^19^

Experimental design RSM in ultrasound-assisted extraction and mean values for the DPPH free radical scavenging assay of *A. minus* flowers are given in Table S2. Antioxidant activity results of the flower extracts were given IC_50_ μg/mL values that were found between 62.48 ± 2.26 and 175.61 ± 0.14 under different extraction conditions. While the maximum IC_50_ value was obtained at 20 °C, 3 min, and 100 W (*R*^2^), the minimum IC_50_ value was obtained at 40 °C, 6 min, and 150 W (R4).

The regression correlation efficiency for the estimated model equation was determined as *R*^2^ = 0.9598 (Table S3). Moreover, since the *p*-value was less than 0.05 and the lack of fit value was greater than 0.05 (not significant), the quadratic model fit this study.

The predicted y response for the IC_50_ value of *A. minus* leaves extracts is expressed in terms of coded values by the following quadratic polynomial equation and eq 2 below:
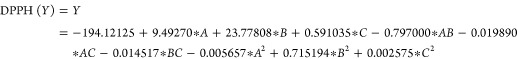
2

According to the analysis variance result, extraction temperature (*A*) (*p* ≤ 0.05), extraction time (*B*) (*p* ≤ 0.05), extraction power (*C*) (*p* ≤ 0.05), correlation effect of extraction temperature and time (*AB*) (*p* ≤ 0.05), and correlation effect of extraction temperature and power (*AC*) (*p* ≤ 0.05) were found to be significant. The regression coefficient was calculated as *R*^2^ = 0.9598, and the corrected coefficient of determination was calculated as *R*_adj_^2^ = 0.9081. A model’s *R*^2^ value must be close to 1.00, the difference between the model’s *R*^2^ and corrected *R*^2^ values must be less than 0.2, and the coefficient of variation (CV) with its very low value of 7.06 indicates that the model is in acceptable fit for experimental data and is reliable. As seen in the equation^20^ regarding the IC_50_ value of *A. minus* leaves samples, the linear term (*B*) of the extraction time has a positive coefficient, which shows that the IC_50_ value increases with the increase of the extraction temperature.

In addition, the fact that this factor has the highest coefficient value shows that it has the most significant effect on the DPPH value.

Experimental design RSM in ultrasound-assisted extraction and mean values for the DPPH free radical scavenging assay of the *A. minus* branch are given in Table S3. Antioxidant activity results of the branch extracts were given IC_50_ μg/mL values that were found between 144.24 ± 3.38 and 338.91 ± 1.23 under different extraction conditions. While the maximum IC_50_ value was obtained at 20 °C, 6 min, and 50 W (R5), the minimum IC_50_ value was obtained at 40 °C, 6 min and 150 W (R6).

The regression correlation efficiency for the estimated model equation was determined as *R*^2^ = 0.9579 (Table S4). Moreover, since the *p*-value was less than 0.05 and the lack of fit value was greater than 0.05 (not significant), the quadratic model fit this study.

The predicted y response for the IC_50_ value of *A. minus* branch extracts is expressed in terms of coded values by the following quadratic polynomial equation and eq 2 below:
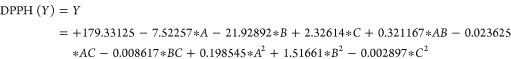
3

According to the analysis variance result, extraction temperature (*A*) (*p* ≤ 0.05), extraction time (*B*) (*p* ≤ 0.05), extraction power (*C*) (*p* ≤ 0.05), quadratic effect of extraction temperature (*A*^2^) (*p* ≤ 0.05), correlation effect of extraction temperature and time (*AB*) (*p* ≤ 0.05), and correlation effect of extraction temperature and power (*AC*) (*p* ≤ 0.05) were found to be significant.

The regression coefficient was calculated as *R*^2^ = 0.9579, and the corrected coefficient of determination was calculated as *R*_adj_^2^ = 0.9039. A model’s *R*^2^ value must be close to 1.00, the difference between the model’s *R*^2^ and corrected *R*^2^ values must be less than 0.2, and the coefficient of variation (CV) with its very low value of 6.05 indicates that the model is in acceptable fit for experimental data and is reliable. As seen in eq 3 regarding the IC_50_ value of *A. minus* branch extracts, the linear term (*C*) of the extraction power has a positive coefficient, which shows that the IC_50_ value increases with the increase of the extraction temperature. In addition, the fact that this factor has the highest coefficient value shows that it has the most significant effect on the IC_50_ value.

**Figure 1:**
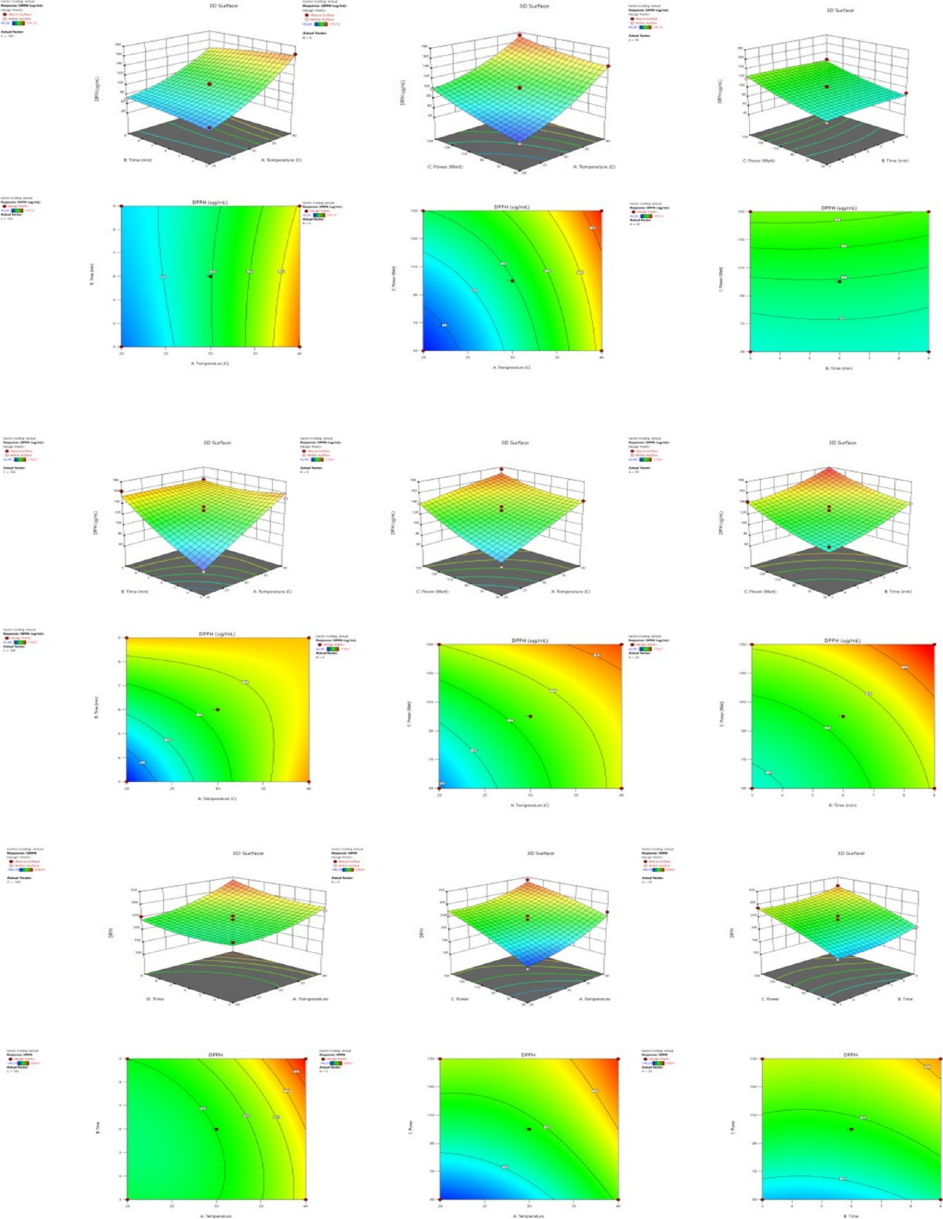
3D response surface and 2D contour drawings showing the effects of temperature (°C), time (min), and ultrasonic power (*W*) on the percentage of extraction efficiency in the extraction of *A. minus* leaves, flowers, and branch extracts.

Three-dimensional (3D) response surface and two-dimensional (2D) contour plots were used to determine the effects of independent variables on the extraction efficiency of *A. minus* leaves, flower, and branch samples (Figure 1). When creating the graphs, the maximum level for the DPPH value was selected, while other factors were kept (at the midpoint of the test range). It showed the interactive effects of extraction temperature, extraction time, extraction temperature-ultrasonic power, and extraction time-ultrasonic power on the IC_50_ value of *A. minus* leaves, flowers, and branch samples.

The IC_50_ values decrease as they differ from the determined parameter values. Based on the results, one of the important factors affecting the IC_50_ value of *A. minus* leaf samples is the extraction temperature; it has been determined that one of the important factors affecting the IC_50_ value of flower samples is extraction time and one of the important factors affecting the IC_50_ value of branch samples is extraction power. Ezzati^21^ reported that, thanks to increased temperature, plant swelling increased, pores in the cell wall expanded, and solvent infusion into the plant matrix was improved. In addition, the release of extracts into the environment is facilitated. On the other hand, it has been determined that as the temperature increases further (extraction temperature >20 °C), deterioration occurs in the structure due to the cavitation effect of ultrasound waves, and therefore, there is a decrease in antioxidant values. The extraction efficiency of *A. minus* leaf, flower, and branch samples was increased by increasing the time for 6 min, but it reached saturation point at a certain temperature and then started to decrease. After this period, excessive exposure to ultrasound and heat treatments caused the antioxidant values to decrease due to the degradation of the obtained extracts in the extraction environment.

### 2.2 Impact of Independent Variables on Ultrasonic-Assisted Extraction by Comparing Antioxidant Potentials

**Figure 2:**
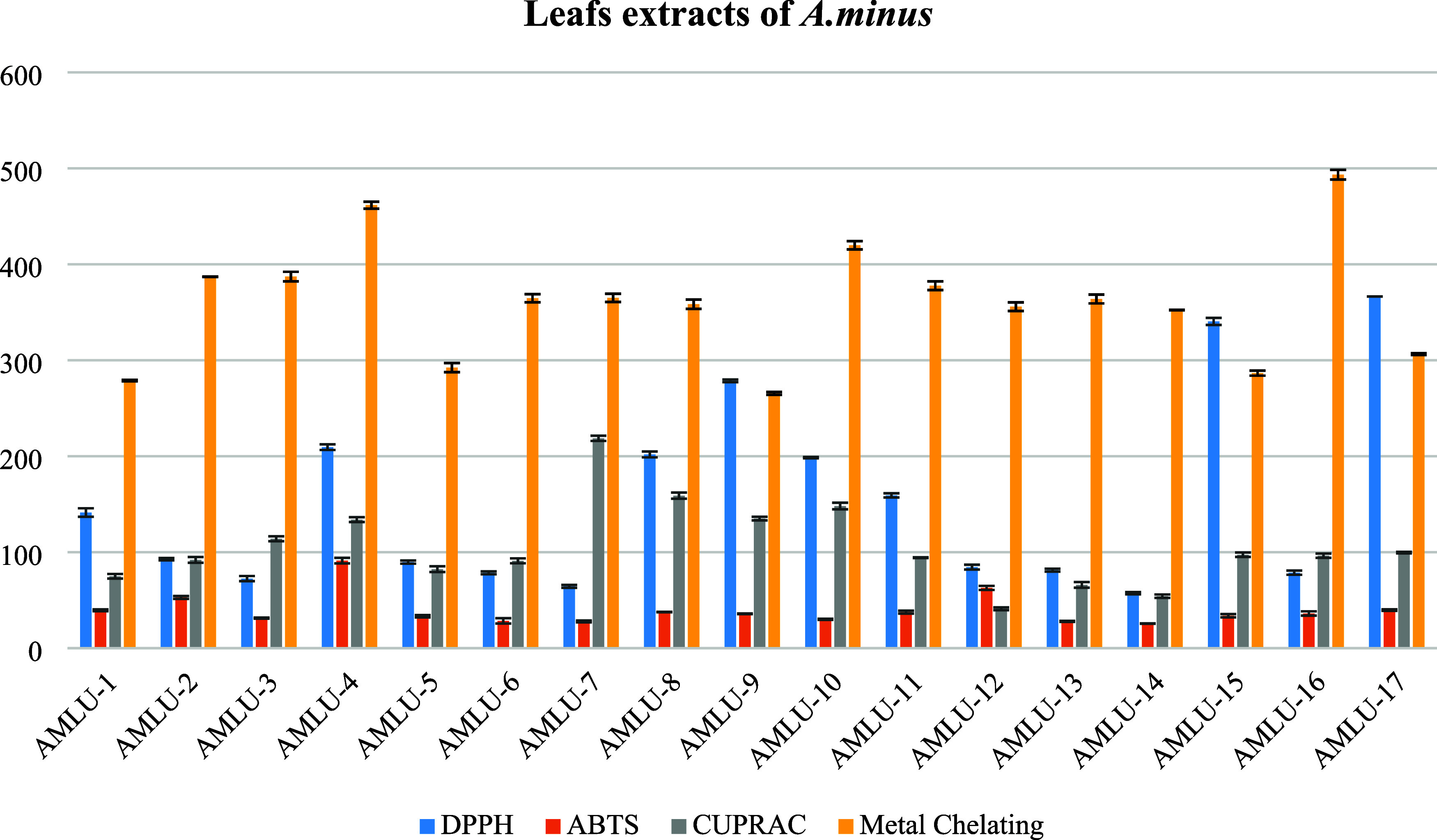
Antioxidant activity results of the leaf extracts obtained by ultrasound-assisted extraction (IC_50_ μg/mL).

The antioxidant activities of obtained 17 extracts through ultrasonic-assisted extraction (UAE) were assessed for each organ of *A. minus* using the DPPH^•^, ABTS^•+^, CUPRAC, and metal-binding methods. The results of antioxidant activity tests were conducted on 17 extracts obtained from *A. minus* leaves. Radical scavenging potential tested by using DPPH free radical and ABTS cation radical assays of the extracts showed better activity than CUPRAC and metal-binding methods (Figure 2). The AMLU-14 extract, which obtained by using among all the extracts, was exhibited strong antiradical activity with IC_50_ values; 55.28 ± 2.21 and 21.80 ± 0.12, respectively, DPPH^•^and ABTS^•+^. Furthermore, the cupric-reducing antioxidant assay of the AMLU-14 extract supported these findings. However, it was observed that the metal-binding activity of all of the extracts exhibited nearly identical potential.

**Figure 3:**
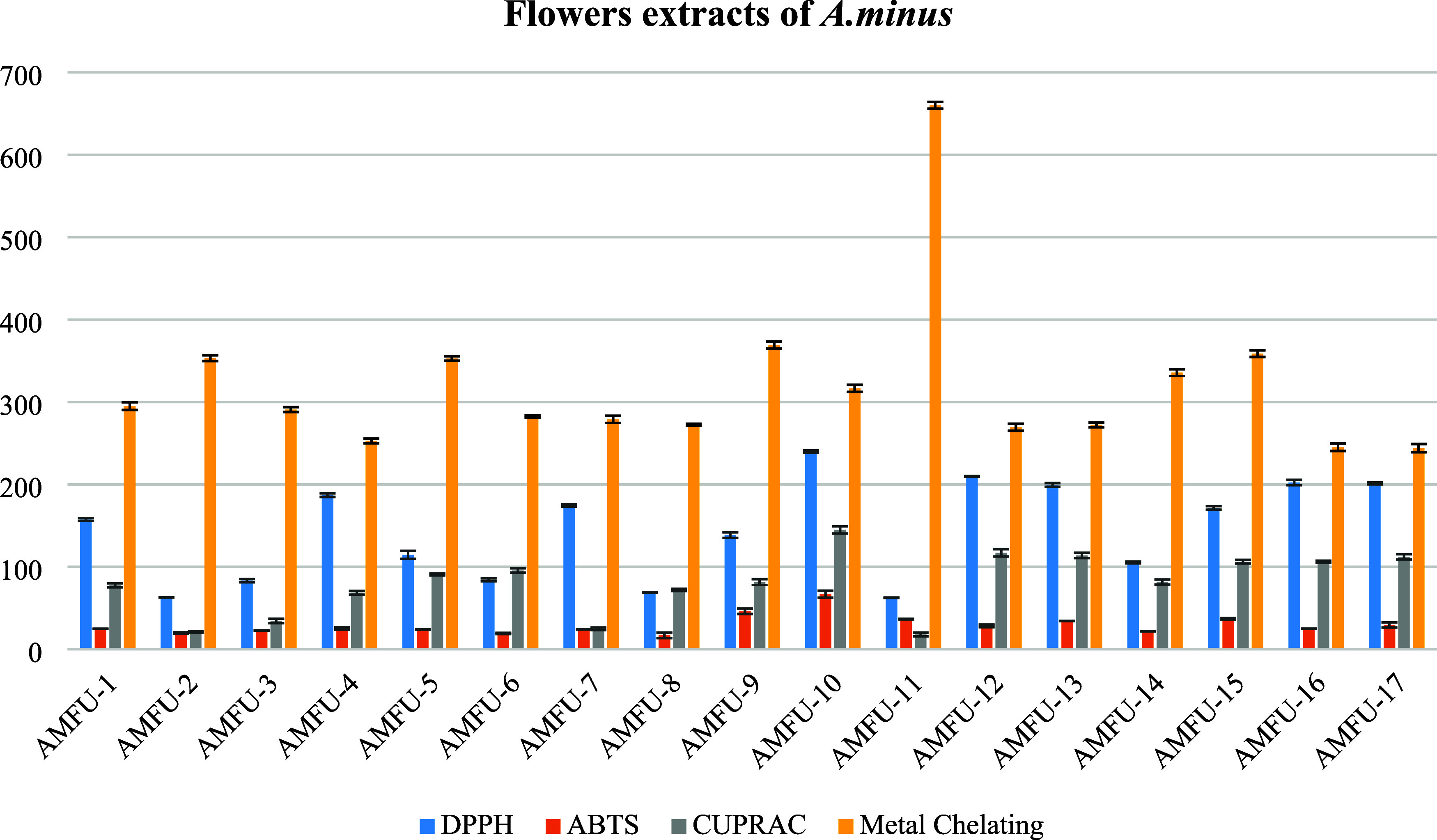
Antioxidant activity results of the flower extracts obtained by ultrasound-assisted extraction (IC_50_ μg/mL).

The flower extracts of the *A. minus* also exhibited strong antioxidant potential (Figure 3). In particular, the AMFU-2 extract exhibited significant antiradical activity when considering the overall evaluation in Figure 3, with IC_50_ values of 62.48 ± 2.26 in the DPPH^•^ scavenging assay and 19.77 ± 0.73 in the ABTS^•+^ scavenging assay. Also, AMFU-8 and AMFU-11 showed almost the same antiradical potential compared with the AMFU-2. Nonetheless, AMFU-2 demonstrated superior cupric-reducing potential compared to AMFU-8, and it also exhibited stronger metal-binding activity than did AMFU-11.

**Figure 4:**
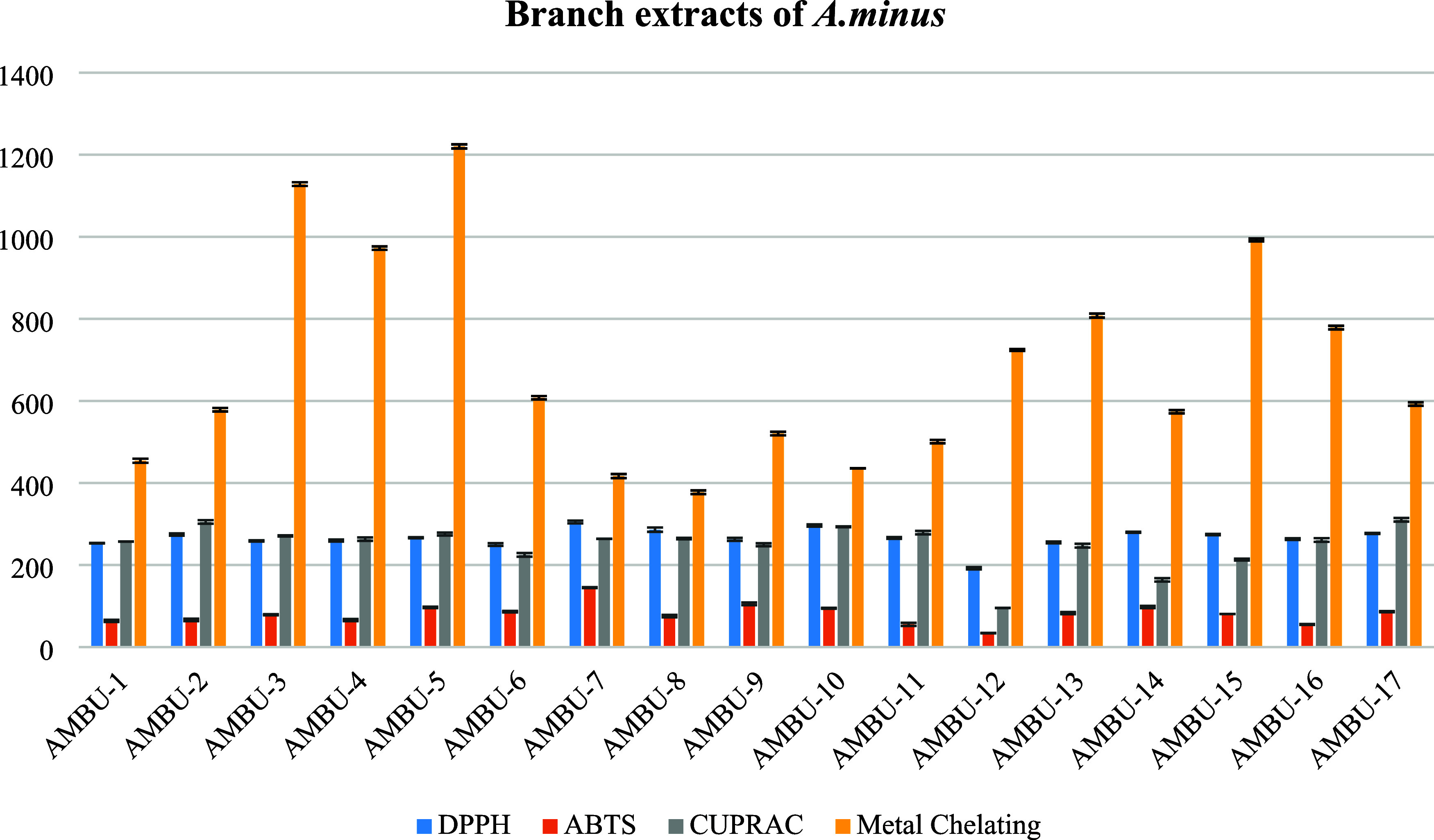
Antioxidant activity results of the branch extracts obtained by ultrasound-assisted extraction (IC_50_ μg/mL).

The branch extracts of *A. minus* also exhibited strong antioxidant potential (Figure 4). In particular, the AMBU-12 extract exhibited significant antiradical activity when considering the overall evaluation in Figure 4, with IC_50_ values of 144.24 ± 3.38 in the DPPH^•^ scavenging assay and 34.07 ± 0.31 in the ABTS^•+^ scavenging assay. Also, AMFU-8 and AMFU-11 showed almost the same antiradical potential with the AMFU-2. Nonetheless, AMFU-2 demonstrated superior cupric-reducing potential compared to AMFU-8, and it also exhibited stronger metal-binding activity than AMFU-11.

### 2.3 Comparison of Ultrasonic Extraction with Conventional Methods by Antioxidant Potentials

**Figure 5:**
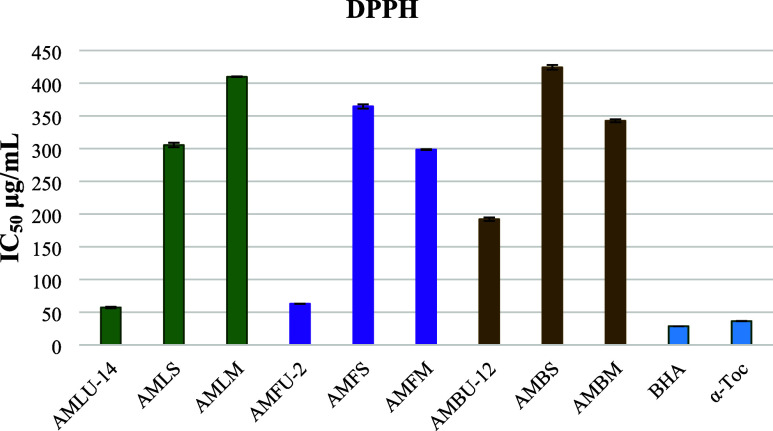
DPPH free radical scavenging activity of the extracts, which were obtained by ultrasound-assisted extraction, Soxhlet extraction, and maceration (IC_50_ μg/mL).

The antioxidant activities of the leaves, flowers, and branches of *A. minus* extracts, obtained through ultrasonic-assisted extraction, Soxhlet, and maceration, were evaluated using DPPH^•^, ABTS^•+^, CUPRAC, and metal chelating methods. The DPPH free radical scavenging activity of the leaf and flower extracts was greater than that of the branch extracts of *A. minus*. Additionally, the AMLU-14 and AMFU-2 extracts, obtained through ultrasonic-assisted extraction, exhibited significantly higher antioxidant activity compared with the AMLS, AMLM, AMFS, AMFM, AMBS, and AMBM extracts, which were obtained using conventional Soxhlet and maceration methods (Figure 5).

**Figure 6:**
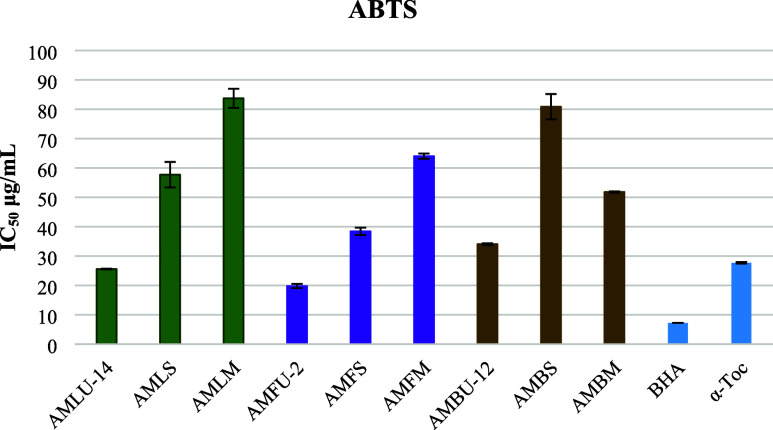
ABTS cation radical scavenging activity of the extracts, which were obtained by ultrasound-assisted extraction, Soxhlet extraction, and maceration (IC_50_ μg/mL).

The ABTS cation radical scavenging activity of the leaf and flower extracts demonstrated nearly identical radical scavenging potential and was superior to that of the branch extracts of *A. minus* (Figure 6). Extracts (AMLU-14, AMFU-2, and AMBU-12) of each organ of the *A. minus* obtained by ultrasonic-assisted extraction showed visibly high activity.

**Figure 7:**
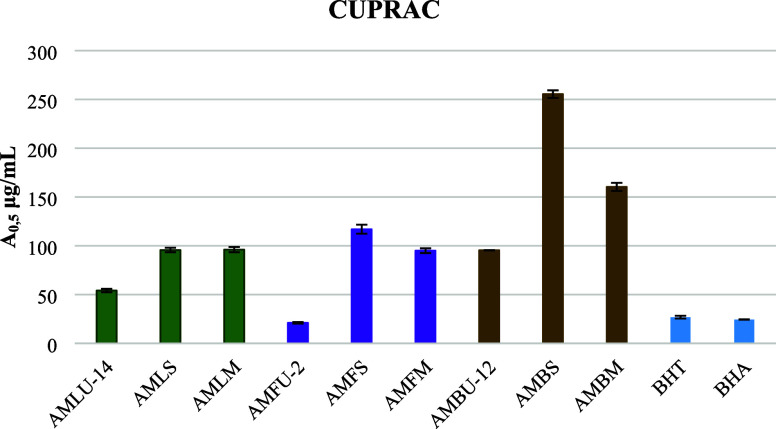
Cupric-reducing antioxidant potential of the extracts, which were obtained by ultrasound-assisted extraction, Soxhlet extraction, and maceration (*A*_0.5_ μg/mL).

Unlike the DPPH^•^ and ABTS^•+^ assays, the cupric-reducing antioxidant potential of the flower extract (AMFU-2) has a great ability to reduce copper(II) ions to copper(I) ions. The extracts obtained through ultrasonic-assisted extraction demonstrated the highest reducing power across all three organs (Figure 7).

**Figure 8:**
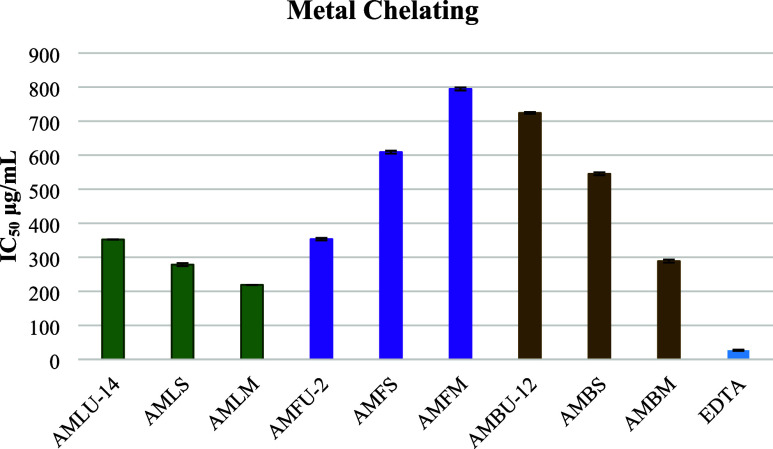
Metal chelating potential of the extracts, which were obtained by ultrasound-assisted extraction, Soxhlet extraction, and maceration (IC_50_ μg/mL).

In contrast to other activities, the metal-binding activity of the extracts obtained by maceration in leaf and branch samples was higher than those obtained by ultrasonic-assisted extraction and Soxhlet. Additionally, among the flower extracts, the one obtained through ultrasonic-assisted extraction exhibited a high metal-binding potential (Figure 8).

### 2.4 Comparison of Ultrasonic Extraction with Other Methods by Phenolic Contents

After identifying the optimum conditions for extracting phenolic compounds from different organs of the *A. minus* using the UAE method, a comparison was conducted to assess the effectiveness of this method against conventional extraction techniques; Soxhlet (SE) and maceration (M). For this purpose, a comprehensive analysis was carried out using liquid chromatography-high resolution electrospray ionization mass spectrometry (LC-HRESIMS) for quantitative measurements.

In total, 26 phenolic compounds were identified in extracts of each organ obtained through the UAE, maceration, and Soxhlet methods. In the analysis of *A. minus* leaves, flowers, and branches, chlorogenic acid is identified as the predominant compound across all parts. The findings are corroborated by a study that identifies chlorogenic acid as the major compound in the leaves of *A. minus* from Kayseri, Türkiye. Additionally, chlorogenic acid was identified as the major compound in a study examining the chemical content of the leaves of *A. lappa*, the most famous *Arctium* species.^22,23^ On the other hand, rutin was determined in each organ of *A. minus* collected from Kars-Türkiye in the current study although it was not defined in any parts of the *A. minus* collected from Kayseri.^17^ Upon a more detailed examination, the specific distribution of the major phenolic compounds is hyperoside and rutin in the leaves,^24^ vanillic acid and apigenin in the flowers, and caffeic acid and vanillic acid in the branch part (Table 2).

**Table 1:** Comparison of Ultrasound-Assisted Extraction, Maceration, and Soxhlet Extraction in Terms of the Phenolic Contents of the Extracts

	leaves (μg/g extract)			flower (μg/g extract)			branch (μg/g extract)		
phenolics	AMLU-14	AMLM	AMLS	AMFU-2	AMFM	AMFS	AMBU-12	AMBM	AMBS
ascorbic acid	29.91	53.10	24.75	54.60	64.92	64.50	26.43	59.28	49.35
chlorogenic acid	24666.96	8492.85	7356.27	1054.92	5200.86	597.99	3501.24	5929.74	1630.02
caffeic acid	442.23	80.22	271.83	164.67	387.42	143.13	115.83	141.36	83.67
(+)-*trans* taxifolin	0.81	0.33	0.24	7.08	6.15	6.78	0.18	1.68	<LOD
luteolin 7-rutinoside	<LOD	<LOD	<LOD	2.31	4.92	2.25	<LOD	<LOD	<LOD
vanilic acid	88.08	207.87	54.99	929.31	2179.86	639.36	166.62	753.51	52.02
luteolin 7-glucoside	4.17	12.12	5.25	87.42	247.77	84.12	1.71	9.72	3.36
rutin	3451.56	371.22	1040.01	43.35	118.44	14.43	36.15	192.42	31.11
hyperoside	4660.20	620.28	2245.35	144.69	411.42	66.87	69.39	489.33	61.92
apigenin 7-glucoside	3.75	11.43	1.80	30.21	44.31	31.74	0.48	2.04	1.95
quercitrin	1299.57	446.43	828.27	43.56	186.84	30.87	34.56	174.09	47.94
quercetin	44.67	3.66	50.07	6.78	12.33	7.53	9.72	4.77	1.95
salicylic acid	95.76	26.46	91.71	52.65	76.89	40.77	90.21	94.83	49.83
naringenin	4.17	84.18	14.10	49.47	36.99	69.45	12.81	24.87	30.39
nepetin	0.18	25.23	2.10	7.89	62.40	15.57	8.43	28.29	1.89
kaempferol	24.42	14.82	69.66	442.95	670.20	576.39	7.35	10.89	14.70
apigenin	2.82	11.16	33.75	689.64	1022.85	904.59	11.67	7.74	22.26
hispidulin	<LOD	22.92	19.92	405.99	563.37	476.43	10.83	19.56	12.84
CAPE	0.45	27.99	2.13	0.27	3.30	2.91	5.58	3.18	15.45
chrysin	3.60	191.34	12.96	3.81	24.93	17.64	35.04	30.30	67.65
acacetin	2.37	96.48	7.11	1.95	12.12	10.11	17.52	12.93	24.06
chrysoeriol	1.02	5.31	7.50	112.44	198.90	130.50	4.65	6.15	4.92
circinor	<LOD	127.83	2.16	<LOD	13.41	7.20	12.27	18.48	29.13
apigenin 7-metilat	0.33	12.48	0.93	0.27	1.50	1.26	2.10	1.62	3.03
3.4-dihydroxybenzaldehyde	2.91	3.39	1.02	1.23	2.40	12.90	2.04	6.63	1.83
pinocembrin	0.33	15.30	1.23	0.21	2.01	1.32	1.86	2.61	2.73

**Table 2:** Lignan Profile of *Arctium* Species in the Literature

lignan	species	parts	ref.
arctiin–arctigenin	A. minus	fruit	(14)
arctiin–arctigenin	A. lappa	fruit	(23,25,28,44,45)
		root	
		stem	
		flower	
arctiin–arctigenin	A. tomentosum	aerial part	(27,46)

### 2.5 Results of the Lignan Content Determination of the Extract by LC-HRESIMS Analysis

**Figure 9:**
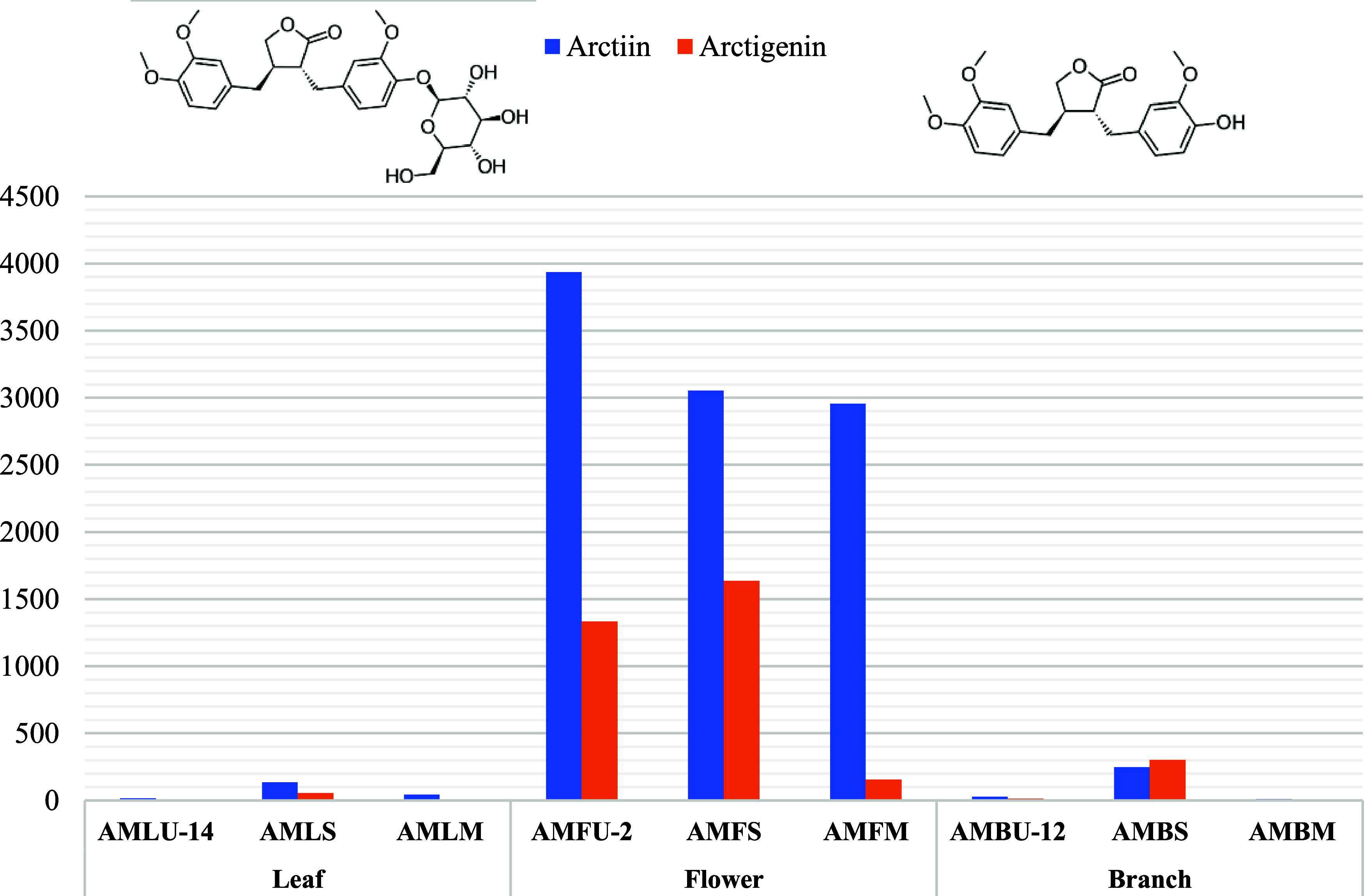
Lignan profile of extracts, which were obtained by ultrasound-assisted extraction, Soxhlet extraction, and maceration from the *A. minus* (μg/g extract).

**Figure 10:**
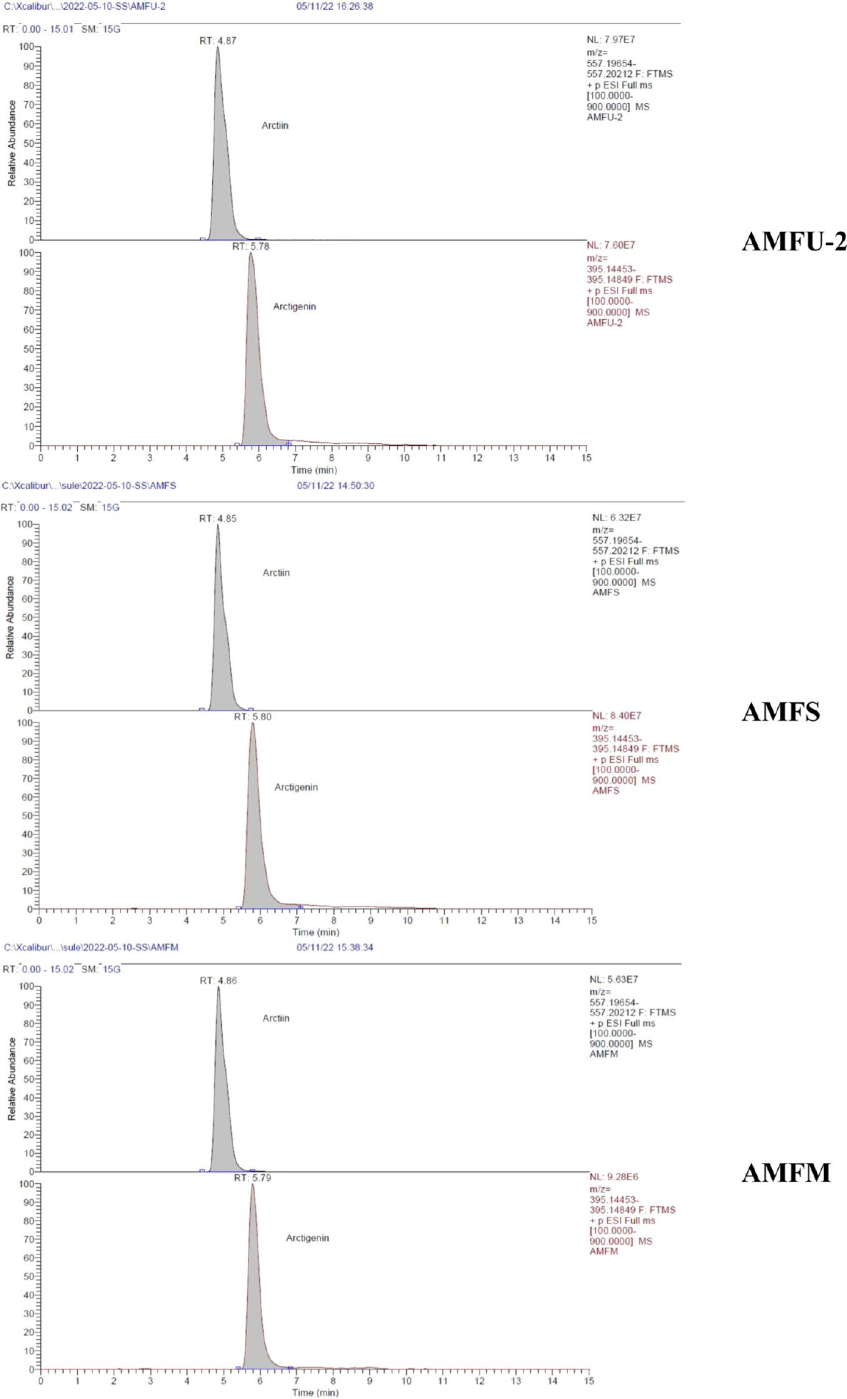
LC-HRESIMS chromatograms of AMFU-2, AMFS, and AMFM flower extracts of *A. minus*.

According to our findings reported for the first time, arctiin (3938.07 μg/g extract) and arctigenin (1332.60 μg/g extract) were quantified to be more abundant in flowers among three separate organs of the *A. minus* by LC-HRESIMS (Figures 9, 10, Table S4). In addition, in all three extraction methods, the content of arctiin was higher than that of arctigenin. However, the only recent study on *A. minus* was collected from Czech Republic showed that the *A. minus* fruits is characterized by a lower content of the major lignan arctiin and a higher content of the aglycone arctigenin. Despite the use of maceration as the extraction method in this study, 96% ethanol was used as the extraction solvent. This choice may have led to a higher concentration of arctigenin in the extract, given its relatively nonpolar structure compared to arctiin.^14^ The biologically active lignans, arctigenin, and its glycoside arctiin are notably abundant in the seeds, roots, fruits, and leaves of both *A. lappa* and *A. tomentosum*.^23,25,26^ The contents of arctiin and arctigenin in *A. tomentosum* Mill. were found to be 10.69 mg/g and 0.15 mg/g, respectively, as determined by high performance liquid chromotagraphy (HPLC).^27^ In the review study where *Arctium* species as well as plants containing arctiin and arctigenin were presented, the ratios of arctiin and arctigenin in *A. lappa* were reported as follows: In fruits, the lignan content (w/w) ranges from 2 to 10% for arctiin and from 0.5 to 2% for arctigenin. In flowers, both arctiin and arctigenin are present at 0.05%. Stems contain 0.06% arctiin, while roots have 0.04% arctiin^28^ (Table 3).

**Table 3:** Cytotoxic Activity Results of the *A. minus* Extracts (IC_50_ μg/mL)^a^

	cell lines						selectivity index			
	HEK293T		MDA-MB-231		HepG2		MDA-MB-231	MDA-MB-231	HepG2	HepG2
	24 h	48 h	24 h	48 h	24 h	48 h	24 h	48 h	24 h	48 h
AMLS	40.50 ± 0.01	6.75 ± 0.01	80.40 ± 0.08	37.50 ± 0.10	471.57 ± 0.01	86.70 ± 0.01	0.50	0.18	0.09	0.08
AMFS	123.20 ± 0.39	8.17 ± 0.17	124.40 ± 0.12	73.60 ± 0.18	178.70 ± 0.02	0.89 ± 0.01	9.93	0.11	0.69	9.18
AMBS	633.40 ± 0.01	215.10 ± 0.21	489.70 ± 0.05	310.10 ± 0.09	921.30 ± 0.03	480.00 ± 0.02	1.29	0.69	0.69	0.45
AMLU-14	11.20 ± 0.15	14.90 ± 0.17	73.00 ± 0.09	177.60 ± 0.14	712.00 ± 0.01	111.00 ± 0.02	0.15	0.08	0.06	0.13
AMFU-2	409.50 ± 0.35	302.00 ± 0.01	96.70 ± 0.10	110.10 ± 0.18	249.10 ± 0.35	12.70 ± 0.19	4.23	2.74	1.64	23.78
AMBU-12	8.24 ± 0.01	31.40 ± 0.01	732.00 ± 0.23	226.40 ± 0.14	18.60 ± 1.85	252.80 ± 0.32	0.01	0.14	0.44	0.12
AMLM	124.30 ± 0.23	33.90 ± 0.18	89.60 ± 0.10	65.10 ± 0.14	54.80 ± 0.28	8.00 ± 0.18	1.39	0.52	2.27	4.23
AMFM	64.60 ± 0.14	80.40 ± 0.15	412.2 ± 0.13	404.90 ± 0.12	388.00 ± 0.04	275.00 ± 0.02	0.16	0.20	0.17	0.29
AMBM	134.00 ± 0.44	>1000	>1000	>1000	571.50 ± 0.04	913.00 ± 0.02			0.23	
arctiin	401.60 ± 0.01	44.60 ± 0.01	723.10 ± 0.01	310.10 ± 0.01	64.50 ± 0.56	1.33 ± 2.84	0.55	0.14	6.22	33.53
arctigenin	140.10 ± 0.01	394.30 ± 0.01	504.00 ± 0.18	206.20 ± 0.24	120.90 ± 0.01	29.50 ± 0.03	0.28	1.91	1.16	13.37

a Data were presented as mean ± standard deviation of individual experiments performed in three parallel measurements (*p* < 0.05).

Also, the effectiveness of the extraction method employed was confirmed in relation to the quantities of arctiin and arctigenin in the obtained extracts. The conclusion drawn was that the ultrasonic-assisted extraction method yielded superior results for both lignans. Moreover, the extract containing the highest arctiin and arctigenin is optimized, and the extraction conditions are determined as 20 °C, 3 min, and 100 W by using response surface methodology.

### 2.6 Results of Cytotoxic Activity Assay

The *in vitro* cytotoxic activity of nine extracts of *A. minus* and two standard compounds (arctiin and arctigenin) was assessed on human healthy cells (HEK293T), human aggresive breast cancer cell line (MDA-MB-231), and human liver cancer cell line (HepG2) (Table 4). When evaluating the cytotoxic effects of the extracts against HEK293T cells, AMFU-2, obtained by ultrasonic-assisted extraction from the flowers of *A. minus*, was found to be the least toxic extract, with an IC_50_ value (>400 μg/mL) comparable to that of arctiin. Moreover, AMFU-2 demonstrated potent inhibition against HepG2 cells, with an IC_50_ value of 12.70 ± 0.19 μg/mL (selectivity index: 23.78) after 48 h of incubation, nearly twice as effective as arctigenin. In addition, AMFS showed the strongest inhibition against HepG2 cancer cells, with an IC_50_ value of 0.89 ± 0.01 μg/mL (selectivity index: 9.18), surpassing the potential of both arctiin and arctigenin, as well as AMFU-2. However, AMFS exhibited a higher cytotoxicity on HEK293T compared to AMFU-2. The only difference between these two extracts is the method of obtaining the extracts, showing that the chemical content is significantly affected by the extraction method. The heat applied during Soxhlet extraction primarily reduced the levels of arctiin and arctigenin (Figure 9).

**Table 4:** Independent Variables and the Levels on Box, Behnken Design

factors	independent variables	unit	factor level		
			–1	0	1
X1	temperature	°C	20	30	40
X2	time	min	3	6	9
X3	power	W	50	100	150

Additionally, it may have caused a decrease in the amounts of chlorogenic acid, rutin, and hyperoside (Table 2), which are major phenolic compounds. A supporting study found that the primary compounds in the fruit of *A. lappa* extract, arctigenin and arctiin, significantly inhibited the proliferation of HepG2 cells.^29^ Moreover, in all flowers and leaves, extracts have the highest cytotoxicity than arctiin and arctigenin against aggressive breast cancer cell (MDA-MB-231). In the cytotoxicity study of the ethanol extract of *A. minus* leaves collected from Canada against aggressive breast cancer cells, the IC_50_ value was determined in sensitive and drug-resistant cancer cell lines 2.93 and 4.53 μg/mL, respectively.^8^

The study indicates that *A. minus* extracts, especially those obtained by ultrasonic-assisted extraction (AMLU-14 and AMFU-2), exhibit significant cytotoxic activities against the studied cell lines. These extracts show potential for use as anticancer agents due to their high efficacy in reducing cell viability. The differential effectiveness across cell lines and extraction methods highlights the importance of further research to optimize extraction techniques and identify the most potent bioactive compounds within *A. minus*.

## 3 Conclusions

This study focused on identifying the bioactive compounds in the leaf, flower, and branch parts of *A. minus* and examined the impact of different extraction methods on these compounds. The antioxidant potential of the extracts was assessed using four different methods with the extracts being obtained through ultrasound-assisted extraction, Soxhlet extraction, and maceration techniques. Also, the cytotoxic effects of these extracts, obtained from various parts of the *A.minus*, were also evaluated against breast and liver cancer cell lines. The results showed that the leaf and flower parts had higher antioxidant activity compared to the branch parts. Moreover, the extracts with the highest antioxidant potential were produced by using the ultrasound-assisted extraction method. The conditions for this extraction method were further optimized using response surface methodology (RSM). And thus, the optimized extraction process proved to be more effective at extracting these antioxidants/anticancers compared to traditional methods such as maceration and Soxhlet extraction.

The ultrasound-assisted extraction, one of the green extraction methods, was used as an extraction method that reduces the extraction solvent, energy consumption, and processing time with an increased extraction efficiency. It was compared with the conventional methods, such as maceration and Soxhlet. The effect of ultrasound conditions on DPPH antioxidant activity in response to 3 different factors (extraction temperature, extraction time, and ultrasonic power) was determined, and the maximum conditions of the factors were determined. In ultrasound-assisted extraction of leaf, flower, and branch extracts of *A. minus*, the minimum IC_50_ value is 20 °C, 6 min, and 50 W for the leaf, 20 °C, 3 min, and 100 W for the flower and 20 °C, 3 min, and 100 W for the branch. In the RSM ultrasonic-assisted extraction modeling of *A. minus* species based on antioxidant potential, the most effective extraction parameter for leaf was temperature; for flower, it was extraction time, and for branch, it was extraction power. AMFU-2 is the extract with the highest amounts of lignans arctiin and arctigenin known for their anticancer properties in the flower part of the plant. The ultrasonic-assisted extraction conditions for this extract have been optimized using RSM. Considering the cytotoxic activity results, it is evident that the ultrasonic-assisted extraction method is highly effective in obtaining concentrated extracts of targeted bioactive compounds. To further enhance the efficiency and applicability of ultrasound-assisted extraction, it is recommended to explore the scalability of this method for industrial applications. Additionally, investigating the synergistic effects of combining ultrasound-assisted extraction with other green extraction techniques could lead to even higher yields and purity of bioactive compounds.

## 4 Materials and Methods

### 4.1 Chemicals and Reagents

2,2-Diphenyl-1-picrylhydrazyl (DPPH), diazanium;3-ethyl-2-[(3-ethyl-6-sulfonato-1,3-benzothiazol-2-ylidene)hydrazinylidene]-1,3-benzothiazole-6-sulfonate (ABTS), ferren, iron, potassium persulfate, copper chloride dihydrate, ammonium acetate, neocuproinin, chloride tetrahydrate, arctiin (Phyto Lab), arctigenin (Phyto Lab), phosphate buffered saline solution (PBS) (PAN-Biotech), ethanol, methanol, dimethylsulfoxide (Isolab), trypan blue (Thermo Fisher Scientific), DMEM/Nutrient Mixture F-12 Ham—with 15 mM Hepes and sodium bicarbonate, w/o l-glutamine, sterile-filtered (Sigma-Aldrich), and fetal bovine serum (FBS), Heat Inactivated, Non-USA Origin Sterile-Filtered, Suitable for Cell Culture (Sigma-Aldrich).

### 4.2 Plant Material

The *A. minus* (Hill) Bernh. was collected from Kars province in Türkiye at an altitude of 1765 m in September 2021. The plant was identified according to the Flora of Turkey and the East Aegean Islands (Kupicha, 1975) by Cagla Kizilarslan Hancer. The scientific name of the identified taxon was updated according to the Worldfloraonline. The voucher specimen was deposited in the Herbarium of Istanbul University, Faculty of Pharmacy (ISTE), Istanbul, Türkiye, with the herbarium no: 117504.

### 4.3 Preparation of the Extracts

#### 4.3.1 Ultrasonic-Assisted Extraction

**Figure 11:**
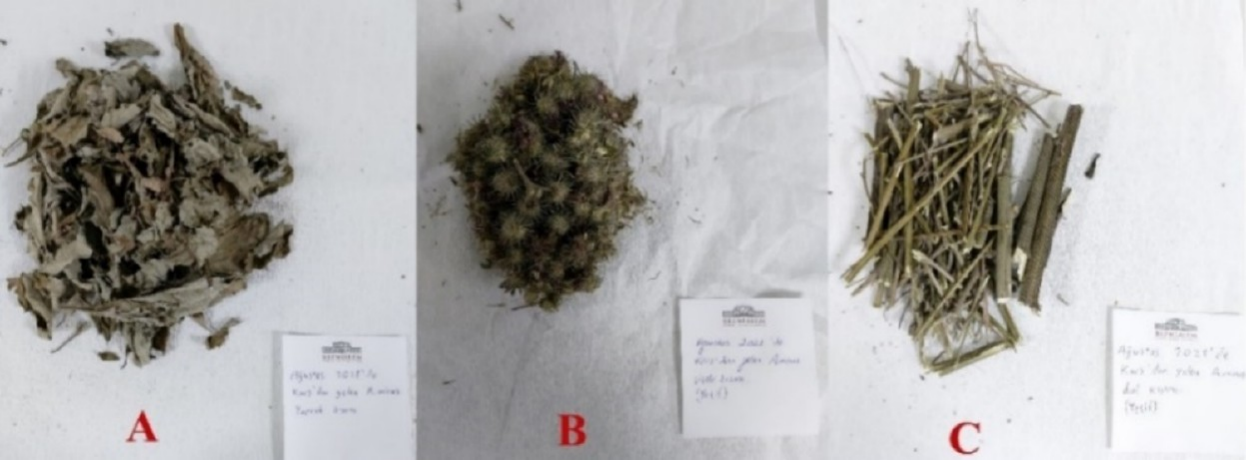
Dried and separated organs of *A. minus* (A: Leaf, B: Flower, and C: Branch; photo created by Cem Taşkın).

The plant was carefully dried in a shaded area under optimal conditions. The leaves, flowers, and branches were then separated, as shown in Figure 11, and subsequently processed using a laboratory-grade blender. A 100 mL portion of methanol was added to 10 g of plant powder in individual tubes and performed in triplicate. These tubes were then placed in a water bath for sonication and exposed to irradiation at different frequencies, with variations in temperature and extraction time as outlined in Table 1. After the extraction process, the mixtures were centrifuged at 5000 rpm for 20 min at 4 °C, and the resulting supernatants were collected. These supernatants were then subjected to vacuum evaporation, and extracts were stored at 4 °C until further use. The extracts obtained from these plant materials were labeled with specific codes, as detailed in Table S1.

##### 4.3.1.1 Response Surface Methodology (RSM) for Ultrasonic-Assisted Extraction

In this study, a methanol solution was used as the solvent. The plant material:solvent ratio was determined as 1:10. The extraction process was carried out with ultrasonic support, and a 3-factor (extraction time, extraction temperature, and ultrasound amplitude) and 3-level response surface methodology (RSM) was used in the optimization study. In the current study, Design Expert Version 7.0.0 software (Stat-Ease Corporation, Minneapolis, MN) was used to create YYM. To evaluate the effect of independent variables, extraction temperature (X1, 20–40 °C), extraction time (X2, 3–9 min), and ultrasonic power (X3, 50–150 W) were determined. In response, a DPPH free radical scavenging assay was determined based on maximum efficiency. The levels of process variables were determined based on preliminary experiments in our laboratory and relevant literature research.^30^ The experimental design of extraction of the *A. minus* leaves was 17 randomized trials with 3 factors coded in terms of experimental design.

Three independent variables are coded as −1 (low level), 0 (medium level), and 1 (high level). In the extraction of leaves, flowers, and branches of the *A. minus* species, the optimization of DPPH free radical scavenging assay data was made according to the RSM in this table.

The regression coefficient and interactions for this model were determined, and the statistical results were evaluated at the 0.05 significance level. Positive signs in the equation show the synergistic effects of the variables, and negative signs show antagonistic effects of the variables. Additionally, the results obtained were used to create 2D contour graphics and 3D graphics (Box and Behnken, 1960). For ultrasound applications, it was implemented with an ultrasonic processor (Hielscher UIP1000, Germany) with a frequency of 20 kHz and a processing power of up to 1000 W. The flow cell of the ultrasonic processor (Hielscher FC100L1K-1S) is combined with the 22 mm diameter probe (Hielscher sonotrode BS4D22).

#### 4.3.2 Maceration

The extraction method involved taking samples of a powdered plant (10 g) and mixing them with 100 mL of methanol for overnight. After the extraction process, the mixtures underwent centrifugation at 5000 rpm × for 20 min at 4 °C. The resulting supernatant was carefully collected, and three extracts (each organ of *A. minus*) were obtained after evaporation of the solvent under the vacuum. Then, all obtained extracts were stored at 4 °C for further utilization.

#### 4.3.3 Soxhlet Extraction

In the Soxhlet extraction (SE), the powdered sample (10 g) is loaded into the Soxhlet extractor. After undergoing a 3 h extraction at 65 °C, the resulting solution was collected and evaporated to be subjected to evaporation to remove the methanol under vacuum conditions. Subsequently, the obtained extracts for each organ were stored at 4 °C for further analysis.

### 4.4 *In Vitro* Antioxidant Assays

#### 4.4.1 DPPH Free Radical Scavenging Assay

The free radical scavenging activity of the extracts was determined by the DPPH^•^ assay.^31^ In brief, a 0.1 mM solution of DPPH in methanol was prepared and then added to sample solutions with 4000, 2000, 1000, 500, 250, 125, 62.5, and 31.25 μg/mL, and then absorbance measured at 517 nm. Antioxidant standards, namely BHA and α-tocopherol, were employed for comparison of activity. The results were reported as the IC_50_ (μg/mL) value, which represents the concentration of the fraction required to achieve a 50% reduction in the specified parameter.^32,33^

#### 4.4.2 ABTS Cation Radical Decolorization Assay

The ABTS^•+^ radical scavenging activities of the extracts were determined according to the literature.^34^ 7 mM of ABTS^•+^ solution was prepared from 2,2-azino-bis(3-ethylbenzothiazoline-6-sulfonic acid) and K_2_S_2_O_3_. The solution was kept in the dark for 24 h at room temperature, and the absorbance of the solution was measured at 734 nm. The solutions of the extracts were prepared in 4000, 2000, 1000, 500, 250, 125, 62.5, and 31.25 μg/mL and added to 96-well plate. Immediately after, the ABTS^•+^ solution was added and incubated at room temperature in the dark. The absorbance of the mixer was measured at 734 nm after 10 min incubation (Each absorbance was taken to be the mean of triplicate measurements). The results were reported as the IC_50_ (μg/mL) value, and BHA and α-tocopherol were used as the standard compound.^35^

#### 4.4.3 CUPRAC Assay

The cupric-reducing antioxidant capacity of the obtained extracts was determined according to the CUPRAC method.^36^ With a few minor adjustments, the procedure described by Apak was utilized to assess the antioxidant activity of the extracts in reducing cupric. The 10 mM Cu^2+^ in the presence of 7.5 mM neocuproine, and NH_4_Ac buffer (1 M, pH 7.0) solutions were mixed with 4000, 2000, 1000, 500, 250, 125, 62.5, and 31.25 μg/mL extract in a 96-well plate. The reduction of Cu^2+^ to Cu^+^ results in the formation of a colored complex, which can be quantified by measuring the absorbance at 450 nm after 1 h incubation at room temperature. The results were calculated as *A*_0.5_ and compared with the absorbance of BHA and α-tocopherol, which were used as antioxidant standards.^37^

#### 4.4.4 Ferrous Ions Chelating Activity

The chelating activity of the extracts on Fe^2+^ was measured using ferrene, with slight modifications. The extracts solution with 4000, 2000, 1000, 500, 250, 125, 62.5, and 31.25 μg/mL concentrations was added to 0.2 mM FeCl_2_. The reaction was initiated by the addition of 0.5 mM ferrene. The mixture was shaken vigorously and left at room temperature for 10 min. After the mixture reached equilibrium, the absorbance was measured at 593 nm. The results were reported as the IC_50_ value, and ethylenediaminetetraacetic acid (EDTA) was used as an antioxidant standard for comparison of the activity.^32^

### 4.5 Quantification of the Phenolic Compounds by LC-HRESIMS Analysis

Orbitrap Q-Exactive HRMS system (Thermo Fisher Scientific Inc., Waltham, MA) was equipped with a heated electrospray ionization source (ESI) operated in positive and negative ionization mode with the HPLC system. In the chromatographic procedure, a Troyasil-C18 reverse-phase column (150 × 3 mm, 5 μm) was employed. Each obtained extract was first dissolved in HPLC-grade methanol, and then, the samples underwent filtration using a syringe filter with a pore size of 0.02 μm. The mobile phases A and B were composed of 1% HCOOH in H_2_O and CH_3_OH, respectively. The gradient program used was as follows: 50% A and 50% B from 0 to 1 min, followed by 100% B from 3 to 6 min, and maintaining 100% B from 7 to 15 min. The flow rate was set at 0.35 mL/min, and the column temperature was maintained at 35 °C. A 1 μL injection volume was used for the analysis.^38−40^

In the high-resolution mode of mass spectrometry analysis (HRMS), the mass spectrometer scanned ions within the *m*/*z* range 100–900. This scanning process generated both negative and positive ions, facilitated using the electrospray ionization source (ESI) as the ionizing agent. The mass spectrometric analysis was configured with the parameters, which were used before.^41^ To quantify the phenolic compounds, a comparison was conducted between the retention times and HRMS data of the sample compounds and established authentic standard compounds. Dihydrocapsaicin was used as an internal standard for LC-HRESIMS measurements.

### 4.6 *In Vitro* Cytotoxic Assay

Human breast cancer (MDA-MB-231), hepatocellular carcinoma (HepG2), and human embryonic kidney (HEK293T) cell lines were purchased from the American Tissue Culture Collection. The cells were cultured in a T75 cell culture flask with a humid environment in a 5% CO_2_ atmosphere at 37 °C in DMEM/F-12 supplemented with 10% FBS and 100 U/mL of penicillin/streptomycin. After the cells reached convenient confluency (80%), the cells were washed with PBS and detached by trypsin/EDTA treatment. Before treatments, 5 × 10^3^ cells were seeded into 96-well plates for 24 and 48 h. The final concentrations of the *A. minus* extracts in the cell cultures were 500, 250, 125, 62.5, 31.25, 15.60, and 7.8 μg/mL. After 24 and 48h of incubation, MTT solution (5 mg/mL in PBS) was added to each well and further incubated at 37 °C with 5% CO_2_ for 4 h in the dark. The cell culture media was aspirated from the wells afterward 100 μL of dimethyl sulfoxide (DMSO) was added into each well to dissolve formazan crystals formed. The absorbance values were measured at 540 nm using an Elisa microplate reader. The experiments were conducted in triplicate, and the results were presented as the mean ± standard deviation.^42,43^

### 4.7 Statistical Analyses

All experiments were conducted in triplicate, and the outcomes were presented as the mean value along with the standard deviation (*p* < 0.05).

## Supporting Information Available

Abbreviations of the extracts, ANOVA results of RSM, experimental design parameters for RSM, table of the lignan content of extracts, and TLC of the extracts (PDF)
