# Supplementary material for: Quantitative Determination of the Cytotoxic Compounds in Different Organs of Arctium minus (Hill) Bernh. by LC-HRESIMS Using Respond Survey Methodology
Source: ACS Omega. 2024 Sep 25;9(40):41890–903. doi: 10.1021/acsomega.4c06644 (PMC11465267; doi:10.1021/acsomega.4c06644)
Supplement: Supplementary file 1 — ao4c06644_si_001.pdf [file ao4c06644_si_001.pdf]

Quantitative Determination of the Cytotoxic Compounds in Different Organs of *Arctium minus* (Hill) Bernh. by LC-HRESIMS using Respond Survey Methodology

Ebru Erol[#], Kubra Feyza Erol[*], Rabia Sare Yanikoglu[§], Cem Taskin[#], Cagla Kizilarslan Hancer[†] and Gulacti Topcu[††, †††]

[#]*Department of Analytical Chemistry, Faculty of Pharmacy, Bezmialem Vakif University, 34093, Istanbul, Türkiye.*
[*]*Department of Nutrition and Dietetics, Hamidiye Faculty of Health Sciences, University of Health Sciences, 34093, Istanbul, Türkiye.*
[§]*Department of Biochemistry, Faculty of Pharmacy, Bezmialem Vakif University, 34093, Istanbul, Türkiye.*
[†]*Department of Pharmaceutical Botany, Faculty of Pharmacy, Bezmialem Vakif University, 34093, Istanbul, Türkiye.*
[††]*Department of Pharmacognosy, Faculty of Pharmacy, Bezmialem Vakif University, 34093, Istanbul, Türkiye.*
[†††]*Drug Application and Research Center, Bezmialem Vakif University, Istanbul, Türkiye.*

Abstract

Arctium minus (Hill) Bernh., commonly known as "Burdock", is a species within the *Arctium* genus of the Asteraceae family. Determining the optimum extraction conditions to obtain a concentrated extract with targeted active ingredients guides the most efficient use of natural products. Herein, ultrasound-assisted extraction (UAE) was optimized using response surface methodology (RSM) to extract bioactive compounds from different organs of *A. minus*. Furthermore, phytochemical composition of extracts of *A. minus* was investigated by using LC-HRESIMS, antioxidant potential by using DPPH, ABTS, CUPRAC, and metal chelation assays, and cytotoxic activities by using human breast cancer cell line (MDA-MB-231) and hepatocellular carcinoma cancer cell line (HepG2), and compared against conventional methods; Soxhlet and maceration. The RSM was employed to investigate the influence of ultrasound-power, extraction-time, and extraction-temperature on the antioxidant potential assessed by the DPPH free radical scavenging assay. In UAE of *A. minus* leaves, flowers, and branches, the conditions resulting in the minimum IC_{50} values: 20°C for 6 minutes at 50 W for leaves, 20°C for 3 minutes at 100 W for flowers, and 20°C for 3 minutes at 100 W for branches. Chlorogenic acid was identified as the major phenolic compound in the extracts obtained by UAE, with concentrations of 24,666.96 µg/g in leaves, 1,054.92 µg/g in flowers, and 3,501.24 µg/g in branches. Flowers of *A. minus* had significantly higher levels of arctiin and arctigenin than leaves and branches. Extracts from leaves and flowers were more effective against MDA-MB-231 and HepG2 cancer cell lines than arctiin and arctigenin.

Keywords: *Arctium minus*, RSM, LC-HRESIMS, Arctiin, Ultrasonic-assisted extraction, Cytotoxicity.
***Corresponding authors.**
e-mail addresses: ebruerol@bezmialem.edu.tr

Table of Contents

**Table S1.**	Abbreviations of the extracts.	2
**Table S2.**	ANOVA results of quadratic model for yield in *A. minus* leaves extracts.	3
**Table S3.**	ANOVA results of quadratic model for yield in *A. minus* flower extracts.	4
**Table S4.**	ANOVA results of quadratic model for yield in *A. minus* branch extracts.	5
**Table S5.**	Experimental design RSM in ultrasound-assisted extraction and mean values for the DPPH free radical scavenging assay of *A. minus* leaves.	6
**Table S6.**	Experimental design RSM in ultrasound-assisted extraction and mean values for the DPPH free radical scavenging assay of *A. minus* flowers.	7
**Table S7.**	Experimental design RSM in ultrasound-assisted extraction and mean values for the DPPH free radical scavenging assay of *A. minus* branch.	8
**Table S8.**	Lignan content of extracts of the *A. minus* (μg/g extract).	8

Figure of Contents

**Figure S1.**	TLC Analysis of the extracts together with arctiin and arctigenin (photo created by Ebru Erol).	9

Table S1. Abbreviations of the extracts.

**Organs & Extraction Method of *A. minus***	**Codes of Extracts**
A. minus Leaves Ultrasonic-1*	AMLU-1
A. minus Flower Ultrasonic-1	AMFU-1
A. minus Branches Ultrasonic-1	AMBU-1
A. minus Leaves Soxhlet	AMLS
A. minus Flower Soxhlet	AMFS
A. minus Branch Soxhlet	AMBS
A. minus Leaves Maceration	AMLM
A. minus Flower Maceration	AMFM
A. minus Branch Maceration	AMBM

*Numerals after the ultrasonic extracts indicate conditions of ultrasonic extraction.

Table S2. ANOVA results of quadratic model for yield in *A. minus* leaves extracts obtained by ultrasound-assisted extraction.

	**Sum of Squares**	**df**	**Mean Square**	**F-value**	***p*-value**	
**Source Model**	18601.28	9	2066.81	59.39	< 0.0013	significant
A-Temperature	*14346.49*	*1*	*14346.49*	*412.26*	*< 0.02103*	
B-Time	*13.24*	*1*	*13.24*	*0.3803*	*0.04569*	
C-Power	*3098.03*	*1*	*3098.03*	*89.03*	*< 0.0164*	
AB	*270.6*	*1*	*270.6*	*7.78*	*0.027*	
AC	*149.08*	*1*	*149.08*	*4.28*	*0.0372*	
BC	*10.99*	*1*	*10.99*	*0.3158*	*0.5917*	
A^2	*582.83*	*1*	*582.83*	*16.75*	*0.0046*	
B^2	*16.96*	*1*	*16.96*	*0.4875*	*0.5076*	
C^2	*97.93*	*1*	*97.93*	*2.81*	*0.1373*	
**Residual**	243.59	7	34.8			
Lack of Fit	*195.04*	*3*	*65.01*	*5.36*	*0.0693*	*not significant*
Pure Error	*48.55*	*4*	*12.14*			
**Cor Total**	18844.88	16				

R^2= 0.9871; R^2 adj= 0.9705; R^2 pred = 0.8304; C. V. % = 5.56; Adequate precision= 7.4186

Table S3. ANOVA results of quadratic model for yield in *A. minus* flower extracts obtained by ultrasound-assisted extraction.

	Sum of Squares	df	Mean Square	F-value	*p*-value	
**Source**						
**Model**	14701.2	9	1633.46	18.57	0.0024	significant
A-Temperature	*4540.09*	*1*	*4540.09*	*51.6*	*0.0012*	
B-Time	*3526.74*	*1*	*3526.74*	*40.08*	*0.0017*	
C-Power	*3564.63*	*1*	*3564.63*	*40.52*	*0.0035*	
AB	*2286.75*	*1*	*2286.75*	*25.99*	*0.0414*	
AC	*395.61*	*1*	*395.61*	*4.5*	*0.0317*	
BC	*18.97*	*1*	*18.97*	*0.2156*	*0.6565*	
A^2	*1.35*	*1*	*1.35*	*0.0153*	*0.9051*	
B^2	*174.45*	*1*	*174.45*	*1.98*	*0.2019*	
C^2	*174.45*	*1*	*174.45*	*1.98*	*0.2019*	
**Residual**	615.88	7	87.98			
Lack of Fit	*490.26*	*3*	*163.42*	*5.2*	*0.8972*	*not significant*
Pure Error	*125.62*	*4*	*31.41*			
**Cor Total**	15317.1	16				

R^2 = 0.9598; R^2 adj = 0.9081; R^2 pred = 0.8751; C. V. % = 7.06; Adequate precision = 6.0770

Table S4. ANOVA results of quadratic model for yield in *A. minus* branch extracts obtained by ultrasound-assisted extraction.

	**Sum of Squares**	**df**	**Mean Square**	**F-value**	***p*-value**	
**Source**						
**Model**	37426.97	9	4158.55	17.72	0.0015	significant
A-Temperature	*12511.25*	*1*	*12511.25*	*53.3*	*0.0132*	
B-Time	*1831.64*	*1*	*1831.64*	*7.8*	*0.0268*	
C-Power	*19453.78*	*1*	*19453.78*	*82.87*	*< 0.0341*	
AB	*371.33*	*1*	*371.33*	*1.58*	*0.0248*	
AC	*558.14*	*1*	*558.14*	*2.38*	*0.0473*	
BC	*6.68*	*1*	*6.68*	*0.0285*	*0.8708*	
A^2	*1659.79*	*1*	*1659.79*	*7.07*	*0.0325*	
B^2	*784.46*	*1*	*784.46*	*3.34*	*0.1103*	
C^2	*220.89*	*1*	*220.89*	*0.941*	*0.3643*	
**Residual**	1643.21	7	234.74			
Lack of Fit	*1320.63*	*3*	*440.21*	*5.46*	*0.0673*	*not significant*
Pure Error	*322.58*	*4*	*80.65*			
**Cor Total**	39070.19	16				

R^2 = 0.9579; R^2 adj = 0.9039; R^2 pred = 0.8463; C. V. % = 6.05; Adequate precision = 6.5180

Table S5. Experimental design RSM in ultrasound-assisted extraction and mean values for the DPPH free radical scavenging assay of *A. minus* leaves.

		**Factor 1**	**Factor 2**	**Factor 3**	**Response 1**
Std	Run	A: Temperature C	B: Time min	C: Power Watt	DPPH ug/mL
11	1	30	3	150	119.25±4.43
12	2	30	9	150	122.57±1.27
9	3	30	3	50	78.19±2.67
17	4	30	6	100	100.75±2.98
7	5	20	6	150	98.46±1.68
16	6	30	6	100	94.78±1.49
13	7	30	6	100	96.35±1.50
6	8	40	6	50	145.37±7.04
14	9	30	6	100	101.25±1.35
8	10	40	6	150	161.13±0.68
2	11	40	3	100	163.852.22
3	12	20	9	100	70.56±5.52
10	13	30	9	50	88.14±3.50
4	14	20	6	50	55.28±2.21
1	15	20	3	100	65.89±6.69
5	16	40	9	100	135.62±1.16
15	17	30	6	100	102.98±0.05

Table S6. Experimental design RSM in ultrasound-assisted extraction and mean values for the DPPH free radical scavenging assay of *A. minus* flowers.

		**Factor 1**	**Factor 2**	**Factor 3**	**Response 1**
Std	Run	A: Temperature °C	B: Time min	C: Power Watt	DPPH ug/mL
15	1	30	6	100	120.16±1.68
8	2	20	3	100	62.48±2.26
16	3	30	6	100	135.28±2.03
1	4	40	6	150	175.61±0.14
11	5	30	3	150	143.91±4.81
14	6	30	6	100	127.53±1.82
10	7	30	9	50	140.18±1.45
5	8	20	6	50	70.23±0.34
2	9	40	3	100	150.09±3.38
4	10	40	9	100	155.43±1.45
6	11	40	6	50	145.62±0.21
12	12	30	9	150	170.38±0.48
3	13	20	9	100	163.46±2.26
9	14	30	3	50	105.56±1.13
7	15	20	6	150	140.23±2.15
13	16	30	6	100	128.18±3.28
17	17	30	6	100	124.72±1.21

Table S7. Experimental design RSM in ultrasound-assisted extraction and mean values for the DPPH free radical scavenging assay of *A. minus* branch.

		**Factor 1**	**Factor 2**	**Factor 3**	**Response 1**
Std	Run	A: Temperature °C	B: Time min	C: Power Watt	DPPH ug/mL
10	1	30	9	50	210.46±0.65
1	2	20	3	100	241.83±2.69
11	3	30	3	150	286.95±1.50
2	4	40	3	100	276.29±2.35
8	5	40	6	150	338.91±1.23
5	6	20	6	50	144.24±3.38
16	7	30	6	100	232.84±3.44
13	8	30	6	100	235.16±5.27
14	9	30	6	100	238.76±6.53
17	10	30	6	100	255.73±2.70
9	11	30	3	50	178.16±2.10
6	12	40	6	50	271.49±4.66
7	13	20	6	150	258.91±4.08
15	14	30	6	100	241.39±1.30
3	15	20	9	100	253.51±1.45
12	16	30	9	150	313.62±4.00
4	17	40	9	100	326.83±1.26

Table S8. Lignan content of extracts of the *A. minus* (μg/g extract).

	**AMLU-14**	**AMLS**	**AMLM**	**AMFU-2**	**AMFS**	**AMFM**	**AMBU-12**	**AMBS**	**AMBM**
**Arctiin**	0,00002	135,75000	44,13000	**3938,07000**	3053,79000	2956,83000	18740,00000	249,18000	10,35000
**Arctigenin**	<LOD	55,56000	<LOD	1332,60000	1636,02000	157,62000	14,58000	302,67000	<LOD

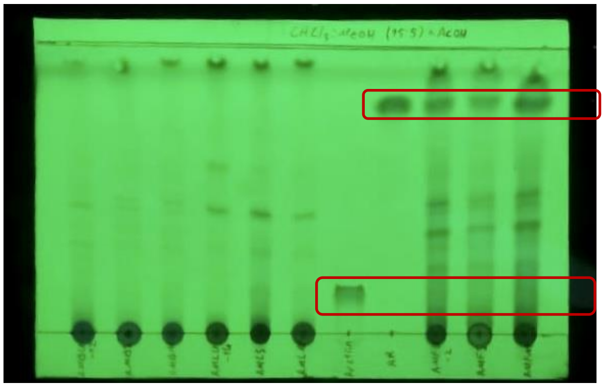

UV-254

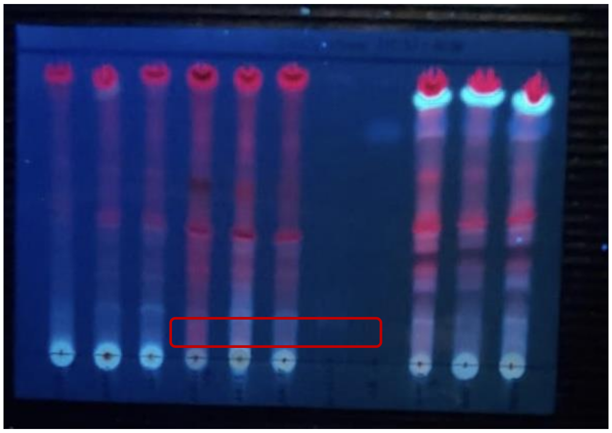

UV-365

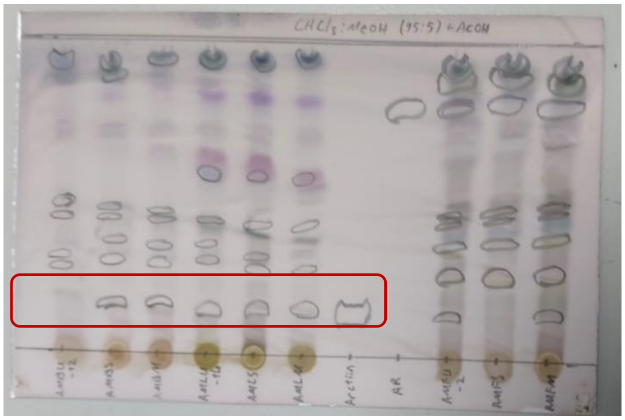

After TLC Spray by using acid

Figure S1. TLC Analysis of the extracts together with arctiin and arctigenin (photo created by Ebru Erol).
